# Impaired HDL2-mediated cholesterol efflux is associated with metabolic syndrome in families with early onset coronary heart disease and low HDL-cholesterol level

**DOI:** 10.1371/journal.pone.0171993

**Published:** 2017-02-16

**Authors:** Timo Paavola, Sanna Kuusisto, Matti Jauhiainen, Sakari Kakko, Tiia Kangas-Kontio, Jari Metso, Pasi Soininen, Mika Ala-Korpela, Risto Bloigu, Minna L. Hannuksela, Markku J. Savolainen, Tuire Salonurmi

**Affiliations:** 1 Department of Internal Medicine, Institute of Clinical Medicine and Biocenter Oulu, University of Oulu, Oulu, Finland and Medical Research Center, Oulu University Hospital, Oulu, Finland; 2 Genomics and Biomarkers Unit, National Institute for Health and Welfare, Biomedicum, Helsinki, Finland; 3 Computational Medicine, Institute of Health Sciences, University of Oulu, Oulu, Finland; 4 NMR Metabolomics Laboratory, School of Pharmacy, University of Eastern Finland, Kuopio, Finland; 5 Oulu University Hospital, Oulu, Finland; 6 Computational Medicine, School of Social and Community Medicine & Medical Research Council Integrative Epidemiology Unit, University of Bristol, Bristol, United Kingdom; 7 Medical Informatics and Statistics Research Group, University of Oulu, Oulu, Finland; 8 Department of Clinical Chemistry, Institute of Diagnostics, University of Oulu, Oulu, Finland; University of Milano, ITALY

## Abstract

**Objective:**

The potential of high-density lipoproteins (HDL) to facilitate cholesterol removal from arterial foam cells is a key function of HDL. We studied whether cholesterol efflux to serum and HDL subfractions is impaired in subjects with early coronary heart disease (CHD) or metabolic syndrome (MetS) in families where a low HDL-cholesterol level (HDL-C) predisposes to early CHD.

**Methods:**

HDL subfractions were isolated from plasma by sequential ultracentrifugation. THP-1 macrophages loaded with acetyl-LDL were used in the assay of cholesterol efflux to total HDL, HDL2, HDL3 or serum.

**Results:**

While cholesterol efflux to serum, total HDL and HDL3 was unchanged, the efflux to HDL2 was 14% lower in subjects with MetS than in subjects without MetS (p<0.001). The efflux to HDL2 was associated with components of MetS such as plasma HDL-C (r = 0.76 in men and r = 0.56 in women, p<0.001 for both). The efflux to HDL2 was reduced in men with early CHD (p<0.01) only in conjunction with their low HDL-C. The phospholipid content of HDL2 particles was a major correlate with the efflux to HDL2 (r = 0.70, p<0.001). A low ratio of HDL2 to total HDL was associated with MetS (p<0.001).

**Conclusion:**

Our results indicate that impaired efflux to HDL2 is a functional feature of the low HDL-C state and MetS in families where these risk factors predispose to early CHD. The efflux to HDL2 related to the phospholipid content of HDL2 particles but the phospholipid content did not account for the impaired efflux in cardiometabolic disease, where a combination of low level and poor quality of HDL2 was observed.

## 1. Introduction

A low HDL-cholesterol (HDL-C) level is a principal epidemiological risk factor for coronary heart disease (CHD) [1]. Although recent Mendelian randomization studies [2,3] have shown that the HDL-C level cannot be considered a causal factor for CHD, certain HDL subpopulations may have a significant atheroprotective role. Plasma HDL consists of various particles with distinct structures and atheroprotective properties [4]. The quantity [5] or quality of these particles may be diversely changed in the low HDL-C level associated cardiometabolic disease and this is not fully revealed by measuring total HDL-C level [6].

There are a number of studies linking cholesterol efflux with cardiometabolic disease. The cholesterol efflux capacity to serum, rather than the macrophage-inherent efflux capacity, has been reported to differ between subjects with low and high HDL-C levels [7]. CHD-patients receiving statin therapy with a normal LDL-cholesterol (LDL-C) level, a low HDL-C level and a high triglycerides level exhibited a lower efflux capacity to serum and to total HDL than healthy subjects [8]. The cholesterol efflux capacity to apoB-depleted serum associated inversely with atherosclerosis and CHD independently of HDL-C or apoA-I levels [9]. Moreover, the risk of future cardiovascular events was inversely associated with the cholesterol efflux capacity to apoB-depleted plasma in a population-based cohort, even after adjusting for traditional cardiovascular risk factors, HDL-C level and HDL particle concentration [10]. Nevertheless, it is still unclear how the cholesterol efflux capacity to the HDL subfractions relates to cardiometabolic diseases.

Here we have studied the functionality of HDL subfractions in cardiometabolic disease. The study samples were derived from Finnish families containing subjects with early CHD in stable condition and a low HDL-C concentration prior to initiation of statin medication as their major risk factor for the disease. The presence of the metabolic syndrome was also common in these families. Cholesterol efflux was measured from cholesterol-loaded THP-1 macrophages to total HDL, HDL2, HDL3 and serum. To provide greater insights into the efflux parameters analyzed we assayed relevant biochemical and enzymatic factors of the HDL fractions and the serum.

## 2. Subjects and methods

### 2.1 Study subjects

The study population (n = 112, Table 1) consisted of Northern Finnish families (n = 24) all of which included a proband with early onset CHD and a low plasma HDL-C level. The inclusion criteria for probands (not for other family members) were plasma HDL-C level below 1.0 mmol/L, LDL-C level below 4.0 mmol/L, triglyceride level below 3.0 mmol/L, no treatment for diabetes and the first CHD event (acute myocardial infarction, coronary angioplasty or coronary bypass operation) before the age of 60. All the inclusion lipid criteria had to be fulfilled in the probands before they started possible statin treatment. Altogether 42 subjects in the entire study population were using statins by the time of blood sample collection and the data in Table 2 is obtained from those blood samples. It is known that statin therapy elevates the HDL-C level [11]. Subjects with a clinical manifestation of CHD, e.g. chest pain or dyspnea, but not fulfilling the previously mentioned criteria for CHD (see above) were defined as unknown with respect to their CHD status (only 2 subjects in the families). Metabolic syndrome was defined based on IDF-criteria [12]. The subjects were collected between 2007 and 2009 in the Oulu University Hospital. A written informed consent was obtained from the subjects. The study was approved by the Ethics Committee of the Oulu University Hospital.

**Table 1:** Clinical characteristics of study subjects.

	Men					Women		
	All	Early CHD ^a^	No CHD ^a^	MetS	No MetS	All	MetS	No MetS
N	58	30	20	33	25	54	25	29
Age (y)	51 (45–57)	52 (49–57)	52 (44–59)	53 (48–57)^e^	49 (32–53)	49 (37–65)	55 (43–67)^d^	45 (32–56)
CHD (n)	31 (53)	30 (100)	0 (0)	23 (70)^f^	8 (32)	8 (15)	6 (24)	2 (7)
Early CHD (n)	30 (52)	30 (100)	0 (0)	22 (67)^f^	8 (32)	5 (9)	4 (16)	1 (3)
CHD age (y)	47 (42–51)	47 (42–51)	NA	48 (43–51)	46 (40–50)	57 (55–66)	57 (53–62)	63 (NA)
MetS (n)	33 (57)	22 (73)	10 (50)	33 (100)	0 (0)	25 (46)	25 (100)	0 (0)
Diabetes (n)	4 (7)	2 (7)	1 (5)	2 (6)	2 (8)	10 (19)	7 (28)	3 (10)
Hypertension (n)	16 (28)	14 (47)^g^	1 (5)	14 (42)^g^	2 (8)	22 (41)	17 (68)^g^	5 (17)
BMI (kg/m^2^)	28.2 (26.1–30.0)	29.5 (27.5–31.2)^e^	27.3 (25.1–28.4)	29.3 (27.9–30.7)^e^	26.3 (23.0–28.2)	27.4 (24.1–31.4)	29.8 (27.4–33.5)^e^	24.8 (22.0–27.5)
Waist (cm)	98 (91–104)	102 (97–107)^e^	95 (88–99)	103 (99–108)^e^	91 (84–95)	89 (81–98)	94 (88–104)^e^	82 (74–91)
Syst.BP (mmHg)	129 (118–140)	130 (115–140)	130 (120–141)	131 (122–141)	123 (114–136)	126 (113–142)	135 (118–143)	122 (111–137)
ACE/ATII (n)	19 (33)	17 (57)^g^	1 (5)	15 (45)^f^	4 (16)	17 (31)	12 (48)^f^	5 (17)
Statin (n)	30 (52)	27 (90)^g^	1 (5)	20 (61)	10 (40)	12 (22)	9 (36)	3 (10)
Smoker (n)	20 (34)	10 (33)	5 (25)	12 (36)	8 (32)	9 (17)	5 (20)	4 (14)
Ex-smoker (n)	24 (41)	16 (53)	7 (35)	15 (45)	9 (36)	8 (15)	3 (12)	5 (17)
Pack-years ^b^	12 (0–28)	23 (9–30)^d^	10 (0–15)	22 (10–32)^e^	5 (0–13)	0 (0–3)	0 (0–2)	0 (0–5)
Alcohol (doses^c^/week)	4 (1–8)	3 (0–6)^d^	5 (2–13)	4 (1–8)	4 (1–10)	1 (0–3)	1 (0–3)	1 (0–5)

Values are expressed as median (interquartile range) or as number of subjects (percentage). Abbreviations: CHD, coronary heart disease; CHD age, the age of the first CHD manifestation; MetS, metabolic syndrome; ACE/ATII, angiotensin converting enzyme inhibitor or angiotensin receptor II blocker medication; waist, waist circumference; syst.BP, systolic blood pressure; NA, not applicable.

^a^ only subjects over 35 years of age included in age-matched groups

^b^ 1 pack-year = 20 cigarettes/day during one year

^c^ dose = 12 g of ethanol; Mann-Whitney U-test between early CHD / no CHD or between MetS / no MetS in sexes separately

^d^ p<0.05

^e^ p<0.01; Pearson chi-square test or Fisher’s test between early CHD / no CHD or between MetS / no MetS in sexes separately

^f^ p<0.05

^g^ p<0.01.

**Table 2:** Plasma/serum biochemical parameters of study subjects.

	Men					Women		
	All	Early CHD ^a^	No CHD ^a^	MetS	No MetS	All	MetS	No MetS
N	58	30	20	33	25	54	25	29
**Lipids and glucose**								
Total-C (mmol/L)	4.40 (3.88–5.20)	3.95 (3.48–4.40)^d^	5.20 (4.93–5.98)	4.40 (3.90–5.30)	4.30 (3.75–5.00)	4.70 (4.10–5.43)	4.70 (3.90–5.80)	4.70 (4.20–5.30)
HDL-C (mmol/L)	1.16 (1.00–1.34)	1.07 (0.85–1.17)^d^	1.28 (1.08–1.67)	1.06 (0.88–1.18)^d^	1.26 (1.17–1.64)	1.50 (1.26–1.69)	1.41 (1.13–1.61)^c^	1.53 (1.35–1.79)
LDL-C (mmol/L)	2.80 (2.28–3.30)	2.40 (1.95–2.90)^d^	3.30 (2.90–4.00)	2.80 (2.30–3.55)	2.80 (1.95–3.15)	2.70 (2.38–3.35)	2.70 (2.35–3.55)	2.70 (2.35–3.40)
TG (mmol/L)	1.32 (0.90–2.13)	1.37 (0.97–2.24)	1.38 (0.63–2.14)	1.87 (1.16–2.31)^d^	0.93 (0.59–1.29)	1.03 (0.80–1.38)	1.34 (1.07–1.76)^d^	0.83 (0.64–1.00)
FFA (mmol/L)	0.44 (0.34–0.55)	0.41 (0.32–0.54)	0.46 (0.35–0.57)	0.44 (0.35–0.55)	0.37 (0.33–0.58)	0.57 (0.46–0.74)	0.57 (0.44–0.74)	0.57 (0.48–0.74)
Glucose (mmol/L)	6.0 (5.5–6.6)	6.3 (5.7–6.7)	5.8 (5.5–6.4)	6.3 (5.8–6.7)^d^	5.5 (5.3–6.4)	5.6 (5.2–6.2)	5.9 (5.7–6.6)^d^	5.3 (5.1–5.6)
HOMA-index ^b^	2.0 (1.3–3.4)	3.2 (2.0–4.5)^d^	1.5 (1.1–2.0)	2.9 (2.0–4.0)^d^	1.3 (0.8–2.0)	2.1 (1.3–3.9)	2.6 (1.8–4.7)^d^	1.4 (1.1–2.7)
**Adiponectins**								
Total (mg/L)	5.99 (4.37–8.92)	5.34 (3.94–7.25)^d^	6.76 (4.85–10.16)	5.46 (4.13–6.42)^d^	7.60 (5.50–10.33)	8.98 (7.27–13.36)	8.23 (6.35–11.94)	9.58 (7.63–14.32)
HMW (mg/L)	1.65 (1.09–3.20)	1.39 (1.08–2.58)	1.91 (1.09–3.98)	1.31 (1.09–2.13)^c^	2.24 (1.22–5.59)	3.11 (2.20–5.29)	2.50 (1.62–4.22)^c^	4.23 (2.39–6.48)
**Apolipoproteins**								
ApoA-I (g/L)	2.05 (1.79–2.31)	1.95 (1.72–2.18)^d^	2.22 (2.00–2.49)	1.98 (1.78–2.27)	2.09 (1.81–2.48)	2.28 (1.95–2.48)	2.29 (1.95–2.50)	2.26 (1.95–2.45)
ApoE in HDL (mg/L)	1.93 (1.41–2.77)	1.70 (1.32–2.30)^c^	2.57 (1.51–4.51)	1.68 (1.19–2.18)^d^	2.50 (1.77–4.40)	3.02 (1.16–4.81)	1.75 (1.01–3.77)	4.32 (1.31–4.97)
**Liver and kidney**								
ALT (U/L)	29 (21–36)	31 (25–41)	27 (20–34)	30 (22–40)	27 (18–33)	20 (15–29)	27 (17–33)^d^	17 (13–24)
Creatinine (μmol/L)	70 (65–74)	69 (61–74)	72 (66–74)	69 (62–74)	70 (68–75)	61 (56–66)	59 (54–67)	61 (59–65)

Values are expressed as median (interquartile range). Abbreviations: Total-C, total cholesterol; HDL-C, HDL-cholesterol; LDL-C, LDL-cholesterol; TG, triglycerides; FFA, free fatty acids; HMW, high-molecular weight.

^a^ only subjects over 35 years of age included in age-matched groups

^b^ see section 2.2.3 for details; Mann-Whitney U-test between early CHD / no CHD or between MetS / no MetS in sexes separately

^c^ p<0.05

^d^ p<0.01.

### 2.2 Methods

#### 2.2.1 Clinical measurements

Smoking history, alcohol intake and medications were obtained from a questionnaire. The lifetime smoking burden was calculated as pack-years (pack-year = 20 cigarettes smoked/day in one year) and alcohol intake was expressed as doses/week (dose = 12 g of 100% alcohol). Waist circumference (cm), height (m) and weight (to the nearest 100 grams) were measured and body mass index was calculated as weight / height squared (kg/m^2^). Blood pressure was measured as a triplicate measurement on the brachium with the Riva-Rocci method.

#### 2.2.2 Blood samples

Blood samples were obtained after an overnight fast (minimum of 8 h). In patients with acute myocardial infarction or coronary bypass operation, the blood samples were taken at least 3 months after acute myocardial infarction or bypass operation. EDTA-plasma and serum samples were obtained by centrifugation at 1500 x g for 15 min at +4C.

#### 2.2.3 Clinical chemistry measurements

Serum insulin concentration and plasma glucose, total cholesterol, LDL-C, HDL-C, triglycerides, free fatty acids and creatinine concentrations and alanine transaminase activity were analyzed in the Laboratory of the Oulu University Hospital. Insulin resistance was calculated as the Homeostatic Model Assessments (HOMA) index i.e. (serum insulin [mU/L] x plasma glucose [mmol/L])/22.5 [13].

#### 2.2.4 Adiponectin measurements

Total adiponectin and high-molecular weight (HMW) adiponectin were measured using a Human adiponectin ELISA kit (Cat # EZHAPD-61K) and a Human HMW-adiponectin ELISA kit (Cat # EZHMWA-64K) supplied by Linco Research Inc. (Missouri, USA).

#### 2.2.5 Isolation and analysis of chemical composition of lipoprotein fractions

Plasma VLDL (d < 1.006 g/mL), LDL (1.019 < d < 1.063 g/mL), total HDL (1.063< d < 1.210 g/mL), HDL2 (1.063 < d < 1.125 g/mL) and HDL3 (1.125 < d < 1.210 g/mL) were isolated by sequential ultracentrifugation [14] using a Beckman ultracentrifuge (Ti 50.4 rotor, 259,000 x g (49,000 rpm), +15°C to isolate total HDL and Ti 50.2 rotor, 218,000 x g (49,000 rpm), +15°C to isolate HDL2 and HDL3). The isolated HDL fractions were immediately dialyzed against PBS and stored at -70°C (without any cryoprotective agent added) prior to the efflux experiments. Isolated lipoprotein fractions were analyzed for lipids as described [15]. To obtain actual plasma concentrations of the lipoprotein associated lipids the loss of lipoproteins in the ultracentrifugation procedure was corrected as described [16].

#### 2.2.6 Analysis of particle size of HDL subfractions

HDL particle size distribution was analyzed by gradient gel electrophoresis as described [17,18]. Self-made 4–26% polyacrylamide native gradient gels (8.0 X 8.0 cm) were used. The stained gels were scanned and analyzed as described [19].

The HDL subfraction distribution was analyzed from the intensity curves using Gaussian model fitting to ensure accurate and consistent subfraction quantifications [20]. The fitting was performed using commercial curve-fitting software (www.perchsolutions.com) allowing for a full optimization of the Gaussian lineshape for each individual intensity curve. The analysis was able to distinguish between the two HDL subfractions, HDL2 and HDL3. The amount of the subfractions was expressed as percentages of total HDL (the sum of both subfractions).

#### 2.2.7 ApoA-I, apoE and preβ-HDL concentrations

Serum apoA-I concentration and the concentration of apoE bound to spherical HDL fraction (measured from ultracentrifuged total HDL fraction) were analyzed by ELISA [21]. Pre-beta-HDL was quantified by crossed immunoelectrophoresis [22]. The pre-beta potential in Table A in S1 Table is defined as the percentage of pre-beta HDL -associated apoA-I of serum total apoA-I after incubating serum for 16 h at 37°C temperature in the presence of LCAT inhibitor.

#### 2.2.8 THP-1 cell culture

Human monocytic cell line THP-1 cells (ATCC #TIB-202, Rockville, MD, USA; obtained from ATCC) [23] were grown on tissue culture treated, 96-well flat-bottom plates with a lid (Costar, Corning Inc, Corning, NY, USA) in RPMI 1640 medium (100 μl) supplemented with 2 mmol/L l-glutamine, 10 mmol/L HEPES, 1 mmol/L Na-pyruvate, 0.05 mmol/L *β*-mercaptoethanol, 4.5 g/L glucose, 100 U/mL penicillin, 0.1 mg/mL streptomycin, and 10% FBS. The cells were further differentiated into mature macrophages by exposure to phorbol 12-myristate 13-acetate (Sigma-Aldrich, St Louis, MO, USA). After 3 days, phorbol 12-myristate 13-acetate containing media were removed and the cells were washed with PBS. FBS-free medium containing radioactive (^3^H-labelled cholesteryl-oleate) acetylated LDL (50 ug/mL as LDL-protein, 2.0 μCi/mL) was added and incubated for 48 hours. Acetylated LDL was produced according to the method of Brown et al. [24]. The cell cultures were maintained in humidified incubators at 37°C in an atmosphere of 5% CO_2_.

#### 2.2.9 Cholesterol efflux measurements

The method used in the cholesterol efflux experiments has been previously described [19,25]. Efflux experiments were performed using 80% to 90% confluent cells in serum-free medium at 37°C in a humidified atmosphere of 5% CO_2_ and 95% air. HDL samples of total HDL, HDL2, and HDL3 (50 μg/mL as protein) and serum samples (0.5% as volume) isolated from the study subjects were used as cholesterol acceptors. An LCAT inhibitor (Na-iodoacetate, I9148 Sigma, 1 mmol/L final) was added to the serum samples used for efflux experiments immediately after their isolation to inhibit LCAT activity and to maintain pre-beta HDL particles in the samples. Each plate also contained a zero sample of plain medium and a control sample (isolated total HDL derived and pooled from several donors in HDL-assays and a control serum sample in serum assay). The samples were incubated for 18 hours after which medium was removed and filtered through a fritted 96 DeepwellTM plates (NUNC, Roskilde, Denmark). The cells were lysed overnight in 0.5 mol/L NaOH. Aliquots of the medium and lysed cells were counted with a beta-counter (Wallac 1414 WinSpectralTM; Perkin Elmer, Wellesley, MA, USA) as a duplicate for radioactivity. Cholesterol efflux was expressed as the percentage of radioactive cholesterol released into the medium from total radioactive cholesterol present in the cells and in the medium per well. Efflux values calculated as medium radioactivity divided by THP-1 cell protein content were correlated with the values calculated as described above. Eight parallel wells were measured for every sample. Individual (i.e. 1–2) outlier values were removed from these eight parallel values in (including zero and control samples) 7 HDL2 samples, 8 HDL3 samples and 9 serum samples. After this, a mean of the parallel values was calculated. In each plate, the mean of the zero sample was subtracted from the mean of each sample, resulting in the final efflux value of a sample. Some of the patient samples (all of them different subjects) were removed from efflux data as erroneous: 9 in HDL2 (3 men with CHD and MetS, 1 man with CHD only, 1 man with MetS only, 2 women with MetS, 2 healthy women); 3 healthy women in HDL3 and 1 man with MetS in serum assay. The intra-assay coefficient of variation of the control samples was 6.9% for total HDL, 3.3% for HDL2, 11.8% for HDL3 and 8.9% for serum assay. In the case of each acceptor (total HDL, HDL2, HDL3, serum), every protocol step was performed during one day (i.e. in one assay) for the entire study population and thus the inter-assay coefficient of variation could not be calculated for single acceptors.

#### 2.2.10 Statistical analysis

Generalized estimating equation model (GEE) [26,27] was used to adjust statistical analyses for the fact that study subjects deriving from a same biological family are biologically related to each other and thus non-independent observations. Pearson, Spearman and partial correlation analyses showed consistent findings compared with GEE models and thus they were used to illustrate correlations. Student’s t-test, Mann-Whitney U-test, Pearson’s chi square test and Fisher’s test were used to show differences in baseline characteristics. Skewed variables were log transformed to obtain the normal distribution for parametric methods. Statistical analysis was conducted with SPSS-software (IBM Corp. Released 2011. IBM SPSS Statistics for Windows, Version 20.0. Armonk, NY, USA). To adjust for multiple testing, p<0.01 was considered statistically significant.

## 3. Results

### 3.1 Study population

The study population (112 subjects from 24 families) contained subjects with premature CHD (30 men and 5 women). Twenty-six of the subjects with premature CHD had also MetS. In addition, some family members without CHD had only MetS or a low HDL-C level. Clinical and biochemical data of the study population are shown in Tables 1 and 2 which include subjects both with and without statin medication. Serum HDL modulating protein parameters and pre-beta HDL forming potential of the clinical groups compared in statistical analyses are shown in Table A in S1 Table. CHD was not studied in females since the number of females with early CHD was too low (Table 1) and the patients were clearly older than women without CHD. Subjects with MetS were older than subjects without MetS (Table 1). Statin medication was used mainly by CHD-affected subjects who also displayed heavier exposure to smoking than subjects not affected by CHD (Table 1). Plasma/serum biochemical parameters were different between the clinical groups (Table 2).

### 3.2 Cholesterol efflux from THP-1 macrophages

The cholesterol efflux data are shown in Table 3. Efflux to HDL2 was 14% higher (p<0.01) in women than in men. No differences between sexes were found in the efflux to the other acceptors. Age was not significantly correlated with the efflux (Table F in S1 Table). No significant correlations were found between the efflux parameters (Table B in S1 Table).

**Table 3:** Cholesterol efflux and HDL2/HDL protein ratio in study subjects.

	Men					Women		
	All	Early CHD ^a^	No CHD ^a^	MetS	No MetS	All	MetS	No MetS
N	58 ^b^	30 ^b^	20 ^b^	33 ^b^	25 ^b^	54 ^b^	25 ^b^	29 ^b^
**Efflux to (%)**								
HDL	9.5 (1.5)^c^	9.6 (1.7)	9.5 (1.3)	9.6 (1.6)	9.4 (1.3)	9.8 (1.7)	10.1 (1.9)	9.6 (1.4)
HDL2 ^b^	10.7 (2.3)^d^	9.8 (2.1)^e^	12.0 (2.3)	9.9 (2.1)^g^	11.7 (2.2)	12.2 (2.4)	11.4 (2.6)^f^	12.9 (2.0)
HDL3 ^b^	14.4 (2.1)^c^	14.1 (2.7)	15.1 (0.9)	14.5 (2.3)	14.3 (2.0)	14.7 (1.8)	14.4 (2.2)	14.9 (1.3)
Serum ^b^	13.6 (2.2)^c^	13.9 (2.4)	13.4 (1.9)	14.1 (2.3)	13.0 (2.1)	13.3 (1.9)	13.3 (2.2)	13.3 (1.7)
**Ratio (%)**								
HDL2/HDL	17.8 (7.6)^d^	15.8 (6.3)	19.8 (8.8)	14.9 (6.1)^g^	21.7 (7.6)	22.7 (8.0)	19.6 (8.1)^g^	25.4 (6.9)

Values are expressed as mean (standard deviation). HDL2/HDL, a ratio of HDL2-protein to total HDL-protein.

^a^ only subjects over 35 years of age included in age-matched groups

^b^ missing efflux values: among men 5 HDL2 and 1 serum efflux (Early CHD 4 HDL2; No CHD 1 HDL2 and 1 Serum; MetS 4 HDL2 and 1 Serum; No MetS 1 HDL2) and among women 4 HDL2 and 3 HDL3 efflux (MetS 2 HDL2; No MetS 2 HDL2 and 3 HDL3); Student’s t-test men vs. women

^c^ p>0.05

^d^ p<0.01; When comparing clinical groups, P-values (p<0.01) of the B_1_-term are reported from two generalized estimating equation models (executed in sexes separately): (1) efflux or HDL2/HDL = B_1_ x MetS + B_2_ x age + intercept, and (2) efflux or HDL2/HDL = B_1_ x early CHD + intercept

^e^ p<0.01 vs. no CHD

^f^ p<0.01 vs. no MetS

^g^ p<0.001 vs. no MetS.

### 3.3 Composition of HDL fractions and their efflux capacity

To pursue more insight into the efflux capacity of the HDL fractions we analyzed the lipid and protein composition of the fractions. The principal correlate with efflux to HDL2 was the ratio of phospholipid content to total protein content in HDL2 particles (Table 4, r = 0.62 in men and r = 0.77 in women, p<0.001 for both). In HDL3 particles, the ratio of phospholipids to total protein was not significantly correlated with their efflux capacity. These associations remained essentially the same after statin using and CHD-affected subjects were removed from the analysis or when confirmed by GEE-models (Tables C-D in S1 Table). In total HDL particles, the ratio of phospholipid content to total protein content was correlated with their efflux capacity in the whole study population (Table 4), but the associations were not significant after excluding CHD-affected or statin using subjects (Tables C-D in S1 Table). Other correlations were either inconsistent among sexes or relatively low.

**Table 4:** Partial correlation coefficients adjusted for age between cholesterol efflux to HDL, HDL2 and HDL3 and ratios of lipid or apolipoprotein E content to protein content in a respective HDL fraction.

	Efflux to					
	HDL		HDL2		HDL3	
	Men	Women	Men	Women	Men	Women
Total cholesterol/protein	0.21	0.40^a^	-0.09	0.01	-0.01	0.11
Free cholesterol/protein	0.09	0.11	0.13	0.12	0.17	0.18
Esterified cholesterol/protein	0.24	0.46^a^	-0.15	-0.04	-0.06	0.06
Triglycerides/protein	0.06	0.27	-0.58^b^	-0.25	-0.16	-0.11
Phospholipids/protein	0.34^a^	0.36^a^	0.62^b^	0.77^b^	0.19	0.09
ApoE/protein	-0.23	-0.19	-	-	-	-

The apoE to protein ratio in HDL and all the composition measures in HDL2 are log-transformed.

^a^ p<0.01

^b^ p<0.001.

When the HDL2 particle phospholipid per protein content was compared in respect to cardiometabolic disease, it was reduced in men with MetS (p = 0.018, Table E in S1 Table) but no other differences were seen.

### 3.4 Association of efflux to HDL2 with MetS and early CHD

Subjects with MetS displayed lower efflux to HDL2 than subjects without MetS (15% lower in men and 12% lower in women, Table 3). The difference was significant after adjustment for sex, age and the HDL2 particle phospholipid per protein content (p = 0.001, Table 5, Table G in S1 Table) and remained similar but not significant after subjects on statin therapy or affected by CHD were removed from the analysis (p = 0.014, Table H in S1 Table). In this subgroup however, MetS was significantly associated with the efflux to HDL2 adjusted only for sex and age, but not for the HDL2 phospholipid per protein content (p = 0.001, Table I in S1 Table).

**Table 5:** Cholesterol efflux to HDL2, early coronary heart disease (CHD) and metabolic syndrome.

	Model	Model predictors	Beta	P-value
Early coronary heart disease and metabolic syndrome	1^a^	**Early CHD** (in men only)	-2.19	<0.001
		HDL2-phospholipids/protein^c^	9.09	0.001
	2^b^	**Metabolic syndrome**	-1.41	0.001
		HDL2-phospholipids/protein^c^	9.48	<0.001
Components of metabolic syndrome	3^b^	**HDL-cholesterol**^d^ (mmol/L)	2.37	<0.001
		HDL2-phospholipids/protein^c^	6.86	0.003
	4^b^	**Triglycerides**^d^ (mmol/L)	-0.83	0.042
		HDL2-phospholipids/protein^c^	9.12	<0.001
	5^b^	**Waist circumference** (cm)	-0.06	<0.001
		HDL2-phospholipids/protein^c^	10.00	<0.001
Other metabolic parameters	6^b^	**VLDL-protein**^d^ (g/L)	-8.55	0.002
		HDL2-phospholipids/protein^c^	8.15	<0.001

Generalized estimating equation model predicting efflux to HDL2

^a^ adjusted for HDL2-phospholipids/protein, age-matched groups are compared in men only

^b^ adjusted for HDL2-phospholipids/protein, age and sex; beta-coefficients denoted as ‘Beta’

^c^ expressed as mmol/L of phospholipids per g/L of protein

^d^ plasma concentrations.

Since efflux to HDL2 was associated with MetS, its relationship to the parameters relating to MetS was studied. The efflux to HDL2 was significantly and positively related to plasma level of HDL-C and negatively with plasma level of VLDL-protein and waist circumference adjusted for sex, age and the HDL2 phospholipid per protein content (Table 5, Table G in S1 Table). These relations remained significant after excluding the subjects receiving statin therapy or affected by CHD (Table H in S1 Table). In addition the plasma levels of total triglycerides and HOMA-index showed inverse associations with the efflux to HDL2 whereas the plasma levels of total- and HMW-adiponectin displayed positive associations (Table 5, Tables G-H in S1 Table). VLDL-particle concentration is inversely related to insulin sensitivity [28] and the VLDL-protein level in Table 5 is reflecting VLDL-particle concentration.

The efflux to HDL2 was 18% lower in men with early CHD as compared with men without CHD (Table 3). The difference was statistically significant adjusted for the HDL2 particle phospholipid per protein content (p<0.001, Table 5). It became statistically non-significant when adjusted for HDL-C level but remained significant when adjusted for other MetS parameters (Table I in S1 Table).

### 3.5 Serum HDL modulating protein parameters, serum potential to form pre-beta HDL particles, clinical patient characteristics and cholesterol efflux

The HDL modulating protein parameters and the pre-beta HDL forming potential of serum were measured to examine whether they affected the efflux to serum (or to HDL fractions), and would this dissipate differences in the efflux parameters between the clinical groups. However, data from our analyses did not support this hypothesis (Tables A and F in S1 Table).

Alcohol intake was not associated with efflux (Table F in S1 Table), whereas smoking men displayed significantly higher efflux to total HDL than non-smoking men (beta-coefficient = 1.01, p<0.01 for smoking status in generalized estimating equation estimating efflux to total HDL with age and smoking status in men). Statins, angiotensin converting enzyme inhibitors and angiotensin receptor II blockers were used mainly by MetS- or CHD-affected subjects and thus their independent effect on the efflux values could not be evaluated.

### 3.6 HDL subfraction distribution in MetS and early CHD

The HDL2/HDL protein ratio was significantly reduced in subjects with MetS in comparison with subjects without MetS when adjusted for sex and age (p<0.001, 31% lower in men and 23% lower in women, Table 3, Table J in S1 Table). When comparing subjects with early CHD and without CHD, this relative reduction of HDL2 in total HDL-protein did not reach statistical significance (Table 3, Table J in S1 Table). An exemplary image of a gel used to separate HDL fractions in electrophoresis is shown in S1 Fig data supplement.

In summary, the HDL2 particle phospholipid per protein content was clearly positively associated with the cholesterol efflux capacity of HDL2 particles, but it did not account for the reduced cholesterol efflux to HDL2 displayed by subjects with cardiometabolic disease in this family population. No evident differences were detected in the other efflux parameters analyzed. In addition, the subjects with MetS displayed a low HDL2/HDL protein ratio in their total HDL.

## 4. Discussion

This study shows an association between the cholesterol efflux to HDL2 and the MetS in a family population, where the low HDL-C level and MetS predispose to early CHD. Supporting associations between the efflux to HDL2 and the relevant metabolic parameters linked with MetS, such as plasma HDL-C level, were detected. The ratio of phospholipids to total protein in the HDL2 particles was the main structural correlate with their efflux capacity. The efflux to HDL2 was not independently associated with premature CHD in men when adjusted for HDL-C level, though it was clearly lower in early CHD-affected men than in men without CHD.

To the best of our knowledge, the cholesterol efflux to HDL subfractions has not been studied in this kind of clinical setting. Tan et al. [29] reported that cholesterol efflux to both HDL2- and HDL3 subfractions was low in subjects with acute coronary syndrome, whereas the CHD-patients in our study were analyzed during their stable period. A similar finding as found in our population was described in a study of obese women after weight loss induced by bariatric surgery [30]. The subjects in that study are metabolically comparable to the affected individuals examined here. The scavenger receptor BI (SR-BI)–mediated efflux to HDL2 was significantly elevated at 6 months after surgery as compared with baseline, whereas the efflux mediated by the ATP-binding cassette transporter G1 (ABCG1) remained unchanged. Efflux to HDL3 displayed no significant change. These findings point to a functional link between obesity and cholesterol efflux to HDL2.

In the present study, the HDL2 particle phospholipid per protein content was correlated with the efflux capacity. The higher phospholipid to protein ratio in HDL particles has been suggested to indicate larger lipoprotein particle size [31]. A relationship between phospholipid content and efflux capacity of HDL particles has been detected both using hepatocytes and macrophages [32–34]. More specifically, the phospholipid content and the size of the HDL particles determine the cholesterol efflux mediated by the SR-BI [35–37]. Also ABCG1 mediated efflux to HDL is correlated with the phospholipid content of HDL particles [38]. With regard to our experimental model of acetyl-LDL loaded human THP-1 macrophages, the ATP-binding cassette transporter A1 (ABCA1) pathway remains predominant, whereas ABCG1 makes only a poor contribution to efflux, and cholesterol removal via the SR-BI pathway is relatively more important [38,39]. Our efflux data are supported by the previous observation from Aron-Wisnewsky et al. [30], where SR-BI–mediated efflux to HDL2 was increased by weight loss, but the question whether the impaired efflux to HDL2 observed in affected subjects in this work was mainly SR-BI -mediated remains to be studied.

We used a well-established and widely utilized in vitro efflux assay [40–42] which measured the efflux of labelled cholesterol from acetyl-LDL loaded THP-1 macrophages to isolated HDL fractions or serum. In our efflux assay using serum as acceptor LCAT-activity was blocked to maintain serum pre-beta HDL particle level. This treatment is different from what has been used in most other whole serum efflux assays. It is noteworthy that the apoB-depleted serum used in previous studies [9,10,43–45] contains enzymes and lipid transfer proteins capable of modulating the metabolic pathways of HDL particles. Li et al. [43] showed that the majority of the cholesterol measured in macrophage cell culture medium (containing apoB-depleted patient serum) after the efflux assay did not reside within HDL particles. Thus, our method using isolated HDL fractions or LCAT-inhibited whole serum as cholesterol acceptors is not comparable to the apoB-depleted serum method.

There are certain limitations in the present study. The probands had *per definition* a low HDL-C level prior to initiation of statin medication as a prominent risk factor for early CHD. Thus, the CHD patients in this population may differ in this respect from CHD patients in the general population. In addition, nearly all of the CHD-affected subjects were receiving statin therapy. There are published reports that men with type 2 diabetes treated with simvastatin and bezafibrate had increased cholesterol efflux to apoB-depleted plasma [46] and pitavastatin treatment has been reported to increase cholesterol efflux to total HDL in dyslipidemic subjects [47]. Accordingly, the statin users in our population might have displayed significantly lower cholesterol efflux parameters prior to statin medication and this might have attenuated the observed differences in the efflux parameters between the clinical groups. Finally, we did not add any cryoprotective agent prior to freezing HDL samples and thus the lipoproteins may have been modified by the freezing/thawing process. However, a study by Kekulawala et al. [48] has shown that cholesterol efflux to HDL samples stored at -20°C for 24 h was not significantly impaired compared with a HDL sample stored at +4°C. Another finding in the study was that the freezing did not alter the size distribution of HDL particles in electrophoresis, regardless of the usage of sucrose as a cryoprotective agent. Our storage temperature for the samples was -70°C.

Only efflux to HDL2 fraction but not to other HDL fractions or serum was impaired in our study. The subjects with MetS and low HDL-C level displayed a so-called ‘double-hit’, i.e. both the low HDL2/HDL ratio and the impaired cholesterol efflux capacity to HDL2. The different HDL2/HDL ratio could in part attenuate a difference in the cholesterol efflux to total HDL fraction or to serum as the low HDL2/HDL ratio implies high HDL3/HDL ratio and the efflux capacity of HDL3 fraction was not impaired. In previous studies, associations between cholesterol efflux to serum and metabolic syndrome have been somewhat diverse, implying either attenuated [7,8] or elevated [44,45] efflux capacity of serum in metabolic syndrome. These paradoxical associations could be partly explained by a high concentration of pre-β HDL particles in serum with simultaneous high triglycerides level [45,49]. Importantly, a detailed experimental protocol and a cell model used for the efflux experiments affect the results observed. Despite our negative result and these previous contradicting reports, sera from subjects with metabolic dyslipidemia or metabolic syndrome may introduce cholesterol accumulation in macrophages once the net cholesterol flux is estimated by measuring cholesterol influx in addition to efflux [44]. At least two hypotheses for the specifically impaired efflux to HDL2 fraction can be considered. First, the HDL3 subfraction has been reported to be more resistant to oxidation than the HDL2 subfraction [50] and thus HDL2 can be oxidized into a dysfunctional form impairing its function in cholesterol efflux [51]. Alternatively, a modified phosphosphingolipidome of HDL in cardiometabolic disease [52] could principally affect the cholesterol efflux to phospholipid-rich HDL2 particles in our efflux assay.

There are at least two ways to interpret the pathological relevance of our findings. The impaired efflux capacity of HDL2 fraction combined with unmodified efflux capacity to total HDL fraction or to serum may have little clinical or biological relevance. On the other hand, the impaired efflux to HDL2 alone could be relevant in vivo if there were, yet unknown, biological effects specific for macrophage cholesterol efflux to HDL2 particles of the cholesterol acceptor spectrum. It is known that cholesterol efflux exerts various biological effects on many cell types [53], and these outcomes are not always uniformly triggered by different apoA-I-containing acceptor particle classes or efflux pathways. As an example, HDL specifically modulates the activity of endothelial nitric oxide synthase by SR-BI-mediated cholesterol efflux [54,55]. SR-BI has also been reported to mediate anti-inflammatory effects of HDL on macrophages although cholesterol efflux has not been shown to mediate these effects [56]. It might turn out that specific lipidomic/proteomic profiling of HDL subclasses is needed to resolve in more detail the particle associated factors affecting HDL–macrophage interaction and capacity of cholesterol efflux.

## 5. Conclusion

The present work demonstrates that the low cholesterol efflux to HDL2 was a clear functional feature of HDL in subjects with a low HDL-C level and derangement of metabolic state predisposing to early CHD. The ratio of phospholipids to total protein in HDL2 particles was positively associated with their cholesterol efflux capacity but the phospholipid content of these particles did not account for the impaired efflux in cardiometabolic disease. In MetS, the impaired efflux to HDL2 co-existed with the reduced protein ratio of HDL2 subfraction to total HDL fraction. The clinical and mechanistic significance of this ‘double-hit’ remains to be elucidated in future studies.

## Supporting information

S1 Text Method description of enzymatic measurements. (DOCX)

S1 Table Tables of supplementary data and analyses. (DOCX)

S1 Dataset Data of study population. (XLSX)

S1 Fig HDL particle size distribution on electrophoresis gel. An exemplary image of five samples.
(PNG)
