# Supplementary material for: Impaired HDL2-mediated cholesterol efflux is associated with metabolic syndrome in families with early onset coronary heart disease and low HDL-cholesterol level
Source: PLoS One. 2017 Feb 16;12(2):e0171993. doi: 10.1371/journal.pone.0171993 (PMC5313225; doi:10.1371/journal.pone.0171993)
Supplement: S1 Text — (DOCX) [file pone.0171993.s001.docx]

**Enzyme measurements**

Serum cholesteryl ester transfer protein (CETP) activity was analyzed by a radiometric method as the transfer/exchange of radiolabeled [^14^C] cholesteryl oleate (Amersham Biosciences) between exogenously added human LDL and HDL, as described previously [1]. Radioactivity in HDL as a measure of transfer activity was determined by liquid scintillation counting. The activity of CETP was expressed as nmol/mL/h. Phospholipid transfer protein (PLTP) activity (nmol/mL/h) and -mass (μg/mL) were determined with radiometric and enzyme-immunoassays as described [2]. Lecithin-cholesterol acyltransferase (LCAT) activity was analyzed by a radiometric assay using a proteoliposome substrate [3]. Briefly, apoA-I -egg lecithin-cholesterol proteoliposome substrate containing radioactive cholesterol tracer was prepared by the cholate dialysis method. The plasma sample and the substrate were incubated at +37C for 30 min after which the LCAT-mediated cholesterol fatty acyl esters were extracted and quantified using thin-layer chromatography for lipid separation and the labelled cholesterol esters were then analyzed for radioactivity. Paraoxonase 1 (PON-1) activity was measured with a chromogenic method [4]. Briefly, in the PON-1 assay, paraoxon (paraoxon-ethyl, D9286-1G, Sigma, St. Louis, MO, USA) was used as the substrate. PON-1 converts paraoxon into *p*-nitrophenol, a yellow compound that can be measured spectrophotometrically at 405 nm. The intra-assay and inter-assay coefficient of variations for PON-1 measurements are 10% and 7%, respectively.

**References**

[1] Groener JE, Pelton RW, Kostner GM. Improved estimation of cholesteryl ester transfer/exchange activity in serum or plasma. Clin Chem. 1986;32: 283-286.

[2] Jauhiainen M, Ehnholm C. Determination of human plasma phospholipid transfer protein mass and activity. Methods. 2005;36: 97-101.

[3] Jauhiainen M, Dolphin PJ. Human plasma lecithin-cholesterol acyltransferase. An elucidation of the catalytic mechanism. J Biol Chem. 1986;261: 7032-7043.

[4] Kleemola P, Freese R, Jauhiainen M, Pahlman R, Alfthan G, Mutanen M. Dietary determinants of serum paraoxonase activity in healthy humans. Atherosclerosis. 2002;160: 425-432.
