# Supplementary material for: Impaired HDL2-mediated cholesterol efflux is associated with metabolic syndrome in families with early onset coronary heart disease and low HDL-cholesterol level
Source: PLoS One. 2017 Feb 16;12(2):e0171993. doi: 10.1371/journal.pone.0171993 (PMC5313225; doi:10.1371/journal.pone.0171993)
Supplement: S1 Table — (DOCX) [file pone.0171993.s002.docx]

**Table A**. Serum pre-beta forming potential, enzyme activities, PLTP activity and PLTP mass in study subjects. Methods of enzymatic measurements are described in S1 Methods.

|  | Men |  |  |  |  | Women |  |  |
| --- | --- | --- | --- | --- | --- | --- | --- | --- |
|  | All | Early CHD ^a^ | No CHD ^a^ | MetS | No MetS | All | MetS | No MetS |
| N | 58 | 30 | 20 | 33 | 25 | 54 | 25 | 29 |
| Pre-beta potential (%) ^b^ | 31.5 (27.0-36.7) | 31.0 (27.0-37.0) | 32.7 (28.6-37.6) | 32.0 (26.3-37.8) | 30.4 (28.0-35.9) | 30.0 (25.4-33.7) | 30.8 (26.2-34.4) | 28.1 (24.9-32.5) |
| PON-1 activity (µmol/min) | 25.3 (15.1-54.6) | 35.3 (22.6-56.8) | 17.6 (13.3-45.0) | 40.2 (15.9-57.7)^d^ | 19.0 (15.1-34.5) | 19.4 (14.7-56.9) | 28.0 (15.6-59.9) | 18.5 (12.8-56.6) |
| PLTP activity (nmol/mL/h) | 6404 (5344-7509) | 6053 (5166-7120) | 6522 (6104-7554) | 6284 (5170-7215) | 6678 (6058-7825) | 6113 (5178-7177) | 6289 (5193-7388) | 5820 (5121-7020) |
| PLTP mass (µg/mL) | 5.99 (4.75-7.16) | 5.23 (4.47-6.69) | 6.21 (5.76-7.32) | 5.49 (4.63-7.11) | 6.27 (5.70-7.31) | 6.76 (5.44-7.63) | 7.11 (6.10-7.79) | 6.40 (5.33-7.69) |
| CETP activity (nmol/mL/h) | 21.9 (17.7-25.5) | 19.1 (16.6-23.1)^c^ | 23.1 (20.8-29.9) | 20.7 (17.0-25.1) | 22.6 (20.1-26.9) | 29.1 (24.5-32.1) | 26.1 (20.4-30.9) | 30.1 (25.6-33.8) |
| LCAT activity (nmol/mL/h) | 36.4 (27.7-43.4) | 38.5 (29.6-47.5)^c^ | 28.6 (20.7-38.8) | 36.4 (25.7-44.7) | 36.4 (28.0-42.0) | 38.8 (31.1-45.4) | 41.0 (30.8-51.2) | 38.4 (31.5-41.2) |

Values are expressed as median (interquartile range). Abbreviations: PON-1, paraoxonase-1; PLTP, phospholipid transfer protein; CETP, cholesterol ester transfer protein; LCAT, lecithin-cholesterol acyltransferase. ^a^ only subjects over 35 years of age included in age-matched groups; ^b^ see section 2.2.7 for details; P-values (<0.05 displayed) of the B-coefficient are derived from a generalized estimating equation model (executed in sexes separately): enzyme value / pre-beta potential = B x early CHD or MetS + intercept: ^c^ p<0.01 vs. no CHD, ^d^ p<0.05 vs. no MetS.

**Table B.** Pearson correlation coefficients between acceptors of cholesterol efflux.

|  | HDL | HDL2 | HDL3 | Serum |
| --- | --- | --- | --- | --- |
| HDL |  | 0.27 | 0.23 | -0.01 |
| HDL2 | *0.20* |  | 0.29 | -0.22 |
| HDL3 | *0.09* | *0.27* |  | -0.14 |
| Serum | *0.08* | *0.10* | *-0.09* |  |

*Values* in italics in the lower left side are for women, and values in the upper right side are for men. No significant (p<0.01) correlations were detected.

**Table C.** Partial correlation coefficients adjusted for age between cholesterol efflux to HDL, HDL2 and HDL3 and ratios of lipid or apolipoprotein E content to protein content in a respective HDL fraction in subjects without statin medication or CHD.

|  | Efflux to | |  |  |  |  |  |  |
| --- | --- | --- | --- | --- | --- | --- | --- | --- |
|  | HDL |  | | HDL2 |  | HDL3 |  |  |
|  | Men | Women | | Men | Women | Men | Women |  |
| Total cholesterol/protein | 0.12 | 0.11 | | 0.05 | -0.05 | 0.00 | 0.18 |  |
| Esterified cholesterol/protein | 0.16 | 0.15 | | 0.03 | -0.09 | -0.14 | 0.09 |  |
| Free cholesterol/protein | -0.01 | -0.01 | | 0.09 | 0.09 | 0.53^a^ | 0.26 |  |
| Triglycerides/protein | -0.02 | 0.16 | | -0.53^a^ | -0.29 | -0.09 | -0.11 |  |
| Phospholipids/protein | 0.27 | 0.16 | | 0.64^a^ | 0.77^b^ | 0.35 | 0.13 |  |
| ApoE/protein | -0.24 | -0.17 | | - | - | - | - |  |

The apoE to protein ratio in HDL and all the composition measures in HDL2 are log-transformed. ^a^ p<0.01, ^b^ p<0.001.

**Table D.** Associations between efflux parameters and phospholipid contents in HDL fractions confirmed by generalized estimating equation models.

|  | Efflux to | | | | | |
| --- | --- | --- | --- | --- | --- | --- |
|  | HDL |  | HDL2 |  | HDL3 |  |
|  | Men | Women | Men | Women | Men | Women |
| **All subjects** |  |  |  |  |  |  |
| Phospholipids/protein ((mmol/L)/(g/L)) | 6.40^a^ | 6.71^a^ | 8.87^a^ | 13.77^b^ | 11.20 | 3.55 |
| **Subjects without statin medication or CHD** | | | | | | |
| Phospholipids/protein ((mmol/L)/(g/L)) | 3.53 | 2.60 | 9.67^b^ | 14.15^b^ | 12.09 | 5.55 |

Model: efflux = B_1_ x phospholipid content + B_2_ age + intercept. B_1_-values and their significance levels are displayed. ^a^ p<0.01, ^b^ p<0.001.

**Table E**. Phospholipid contents of HDL fractions in clinical groups compared.

|  |  | Men |  |  |  |  | Women |  |  |
| --- | --- | --- | --- | --- | --- | --- | --- | --- | --- |
|  |  | All | Early CHD | No CHD | MetS | No MetS | All | MetS | No MetS |
| **All subjects** | N | 58 | 30 | 20 | 33 | 25 | 54 | 25 | 29 |
|  | HDL | 0.60 (0.57-0.66) | 0.59 (0.56-0.63) | 0.60 (0.54-0.69) | 0.59 (0.54-0.62)^a^ | 0.63 (0.58-0.70) | 0.70 (0.63-0.76) | 0.68 (0.62-0.75) | 0.72 (0.64-0.76) |
|  | HDL2 | 0.97 (0.89-1.09) | 0.95 (0.87-1.04) | 0.94 (0.86-1.11) | 0.94 (0.85-1.00)^a^ | 1.04 (0.93-1.12) | 1.08 (0.97-1.13) | 1.03 (0.95-1.12) | 1.10 (1.02-1.16) |
|  | HDL3 | 0.58 (0.55-0.61) | 0.58 (0.54-0.60) | 0.59 (0.56-0.61) | 0.58 (0.55-0.60) | 0.60 (0.56-0.61) | 0.59 (0.57-0.64) | 0.61 (0.58-0.65) | 0.58 (0.57-0.61) |
| **Subjects without statin medication or CHD** | N | 25 | - | - | 10 | 15 | 41 | 16 | 25 |
|  | HDL | 0.60 (0.56-0.70) | - | - | 0.58 (0.52-0.65) | 0.65 (0.58-0.73) | 0.71 (0.62-0.75) | 0.68 (0.61-0.74) | 0.72 (0.63-0.76) |
|  | HDL2 | 0.99 (0.88-1.10) | - | - | 0.88 (0.80-1.03) | 1.07 (0.94-1.12) | 1.06 (0.97-1.16) | 1.03 (0.97-1.12) | 1.10 (0.99-1.16) |
|  | HDL3 | 0.58 (0.56-0.61) | - | - | 0.58 (0.56-0.63) | 0.60 (0.55-0.61) | 0.59 (0.57-0.62) | 0.60 (0.57-0.64) | 0.59 (0.58-0.62) |

Contents are expressed as a ratio of phospholipid concentration to protein concentration (mmol/L to g/L) as median (interquartile range). P-values of the B-coefficient are derived from generalized estimating equation model (executed in sexes separately): phospholipid/protein ratio = B x early CHD or MetS + intercept: ^a^ p<0.05, ^b^ p<0.01.

**Table F.** Correlation coefficients between cholesterol efflux, clinical and metabolic variables, serum HDL modulating protein parameters, serum pre-beta potential and apolipoprotein levels.

|  | Efflux to | | | | | | | |
| --- | --- | --- | --- | --- | --- | --- | --- | --- |
|  | HDL | | HDL2 | | HDL3 | | Serum | |
|  | Men | Women | Men | Women | Men | Women | Men | Women |
| Age | 0.16 | 0.11 | 0.16 | 0.18 | 0.21 | 0.22 | 0.22 | -0.03 |
| Pack-years | 0.26^e^ | -0.03 | -0.20 | -0.23 | -0.03 | -0.08 | 0.29^e^ | -0.19 |
| Alcohol intake | -0.03 | -0.03 | 0.01 | 0.09 | -0.02 | -0.12 | 0.20 | 0.00 |
| ALT | 0.23 | 0.01 | -0.18 | -0.30 | -0.33 | 0.16 | -0.10 | 0.01 |
| Creatinine | -0.09 | 0.00 | 0.03 | 0.17 | -0.05 | 0.05 | -0.04 | -0.04 |
| Waist | 0.22 | -0.10 | -0.39^c^ | -0.35 | -0.14 | -0.21 | 0.21 | -0.06 |
| Body mass index | 0.26 | -0.13 | -0.39^c^ | -0.26 | -0.13 | -0.23 | 0.11 | 0.03 |
| Glucose ^a^ | 0.11 | -0.16 | -0.04 | -0.20 | -0.04 | 0.04 | 0.04 | -0.13 |
| Insulin ^a^ | 0.09 | -0.01 | -0.38^c^ | -0.31 | -0.11 | -0.05 | 0.17 | 0.00 |
| HOMA-index ^ab^ | 0.10 | -0.05 | -0.33 | -0.31 | -0.11 | -0.03 | 0.15 | -0.04 |
| Adiponectin ^a^ | -0.21 | -0.38^c^ | 0.52^d^ | 0.28 | 0.05 | -0.10 | -0.18 | 0.15 |
| HMW-adiponectin ^a^ | 0.00 | -0.31 | 0.50^d^ | 0.34 | 0.11 | 0.06 | -0.10 | 0.19 |
| HDL-cholesterol ^a^ | 0.01 | -0.06 | 0.76^d^ | 0.56^d^ | 0.08 | -0.01 | -0.19 | 0.02 |
| Triglycerides ^a^ | 0.08 | 0.05 | -0.50^d^ | -0.43^c^ | -0.10 | -0.19 | 0.48^d^ | 0.19 |
| VLDL-cholesterol | 0.11 | 0.00 | -0.44^c^ | -0.50^d^ | -0.04 | -0.31 | 0.31 | 0.06 |
| VLDL-triglycerides | 0.12 | -0.06 | -0.50^d^ | -0.57^d^ | -0.05 | -0.25 | 0.35^c^ | 0.04 |
| VLDL-protein | -0.01 | -0.14 | -0.57^d^ | -0.58^d^ | 0.00 | -0.27 | 0.39^c^ | 0.05 |
| LDL-cholesterol | -0.12 | -0.08 | -0.10 | -0.28 | 0.01 | -0.18 | 0.24 | 0.13 |
| ApoA-I | 0.09 | 0.32 | 0.60^d^ | 0.45^c^ | 0.05 | 0.24 | -0.04 | 0.19 |
| ApoE in HDL ^a^ | -0.27 | -0.25 | 0.59^d^ | 0.31 | 0.15 | 0.12 | -0.30 | 0.21 |
| HDL2/HDL ratio ^b^ | 0.17 | 0.03 | 0.63^d^ | 0.64^d^ | 0.16 | 0.04 | -0.23 | 0.06 |
| Pre-beta potential ^b^ | -0.14 | -0.15 | 0.06 | -0.19 | 0.16 | -0.06 | 0.05 | -0.29 |
| PLTP activity | 0.13 | 0.17 | 0.03 | 0.14 | -0.36^c^ | -0.30 | 0.09 | 0.13 |
| PLTP mass | 0.03 | 0.15 | 0.44^c^ | 0.48^c^ | -0.33 | -0.07 | -0.06 | -0.36 |
| CETP activity | -0.12 | 0.00 | 0.36^c^ | 0.09 | -0.09 | -0.22 | -0.15 | 0.05 |
| LCAT activity | -0.06 | 0.43^c^ | 0.01 | 0.11 | -0.31 | -0.10 | 0.11 | 0.09 |
| PON1 activity ^a^ | 0.07 | -0.17 | -0.08 | -0.04 | -0.05 | 0.19 | -0.16 | 0.14 |

Biochemical variables are plasma/serum concentrations. Abbreviations: Waist, waist circumference, HMW, high-molecular weight; PON-1, paraoxonase-1; PLTP, phospholipid transfer protein; CETP, cholesterol ester transfer protein; LCAT, lecithin-cholesterol acyltransferase. Pearson’s correlation coefficient in ‘Age’, Spearman’s rho in ‘Pack-years’ and ‘Alcohol intake’ and partial correlation coefficient adjusted for age in other variables. ^a^ log-transformed; ^b^ see section 2.2 for details; ^c^ p<0.01, ^d^ p<0.001; only in ‘Age’, ‘Pack-years’ and ‘Alcohol intake’: ^e^ p<0.05.

**Table G.** Cholesterol efflux to HDL2 predicted by metabolic syndrome and its parameters.

|  | Model | Model | Men |  | Women |  |
| --- | --- | --- | --- | --- | --- | --- |
|  | number | predictor | Beta | P-value | Beta | P-value |
|  | 1 | **Metabolic syndrome** | -2.51 | <0.001 | -1.98 | 0.002 |
| Parameters of metabolic syndrome | 2 | HDL-cholesterol (mmol/L)^a^ | 4.43 | <0.001 | 3.22 | <0.001 |
|  | 3 | Triglycerides (mmol/L)^a^ | -1.48 | <0.001 | -2.24 | <0.001 |
|  | 4 | VLDL-protein (g/L)^a^ | -13.14 | <0.001 | -17.62 | <0.001 |
|  | 5 | Adiponectin (mg/L)^a^ | 0.32 | <0.001 | 0.10 | 0.065 |
|  | 6 | HMW-adiponectin (mg/L)^a^ | 0.51 | <0.001 | 0.15 | 0.080 |
|  | 7 | HOMA-index | -0.02 | 0.362 | -0.05 | 0.018 |
|  | 8 | Waist circumference (cm) | -0.09 | 0.008 | -0.08 | 0.002 |

Abbreviations: HMW, high-molecular weight. Model: efflux to HDL2 = B_1_ x predictor + B_2_ x age + intercept. B_1_ and its p-value are reported; ^a^ plasma concentration.

**Table H**. Cholesterol efflux to HDL2 predicted by metabolic syndrome and its parameters in subjects without statin medication or CHD.

|  | Model number | Model predictor | Beta | P-value | Model number | Model predictors | Beta | P-value |
| --- | --- | --- | --- | --- | --- | --- | --- | --- |
|  | 1 | Metabolic syndrome | -0.86 | 0.001 | 6 | Metabolic syndrome  HDL2-PL/protein^a^ | -0.45  0.63 | 0.014  <0.001 |
| Parameters of metabolic syndrome | 2 | HDL-cholesterol^ab^ | 0.63 | <0.001 | 7 | HDL-cholesterol^ab^  HDL2-PL/protein^a^ | 0.29  0.50 | 0.004  <0.001 |
|  |  |  |  |  |  |  |  |  |
|  | 3 | Triglycerides^ab^ | -0.43 | <0.001 | 8 | Triglycerides^ab^  HDL2-PL/protein^a^ | -0.11  0.64 | 0.298  <0.001 |
|  | 4 | VLDL-protein^b^ | -0.57 | <0.001 | 9 | VLDL-protein^b^  HDL2-PL/protein^a^ | -0.24  0.54 | 0.003  <0.001 |
|  | 5 | Waist circumference | -0.53 | <0.001 | 10 | Waist circumference  HDL2-PL/protein^a^ | -0.32  0.61 | <0.001  <0.001 |

HDL2-PL/protein, a ratio of phospholipid concentration to protein concentration in HDL2. Models 1-5: efflux to HDL2 = B_1_ x predictor + B_2_ x age + intercept; Models 6-10: efflux to HDL2 = B_1_ x predictor + B_2_ x age + B_3_ x HDL2-PL/protein + intercept. B-coefficients and their p-values are reported. All the continuous variables (or their log-transformations) except age are transformed to z-scores in sexes separately to adjust analyses for sex. ^a^ log-transformed; ^b^ plasma concentration.

**Table I**. Cholesterol efflux to HDL2 predicted by early coronary heart disease (CHD), cardiovascular risk factors and metabolic syndrome parameters in men.

| Model number | Model predictors | Beta | P-value |
| --- | --- | --- | --- |
| 1 | Early CHD | -2.21 | 0.001 |
| 2 | Early CHD  Smoker (yes/no) | -2.07  -1.12 | 0.001  0.101 |
| 3 | Early CHD  LDL-cholesterol (mmol/L)^a^ | -2.80  -0.77 | <0.001  0.124 |
| 4 | Early CHD  HDL-cholesterol (mmol/L)^a^ | -0.37  4.57 | 0.483  <0.001 |
| 5 | Early CHD  Triglycerides (mmol/L)^a^ | -1.93  -1.48 | <0.001  <0.001 |
| 6 | Early CHD  VLDL-protein (g/L)^a^ | -1.77  -12.91 | <0.001  <0.001 |
| 7 | Early CHD  Adiponectin (mg/L)^a^ | -1.62  0.26 | 0.007  <0.001 |
| 8 | Early CHD  HMW-adiponectin (mg/L)^a^ | -1.76  0.45 | 0.005  <0.001 |
| 9 | Early CHD  HOMA-index | -2.23  0.01 | 0.003  0.929 |
| 10 | Early CHD  Waist circumference (cm) | -1.97  -0.02 | 0.008  0.490 |

Abbreviations: HMW, high-molecular weight. Efflux to HDL2 is predicted in men over 35 years (to produce age-matched groups of 26 early CHD-patients and 19 subjects without CHD) by different generalized estimating equation models: efflux to HDL2 = B_1_ x early CHD + B_2_ x other predictor + intercept. B-coefficients and their p-values are reported. ^a^ plasma concentration.

**Table J.** HDL2/HDL ratio predicted by early coronary heart disease (CHD) or metabolic syndrome (MetS).

|  | HDL2/HDL ratio in HDL ^a^ | |
| --- | --- | --- |
|  | Beta | P-value |
| Early CHD ^b^ | -4.0 | 0.074 |
| Metabolic syndrome |  |  |
| Men ^c^ | -8.8 | <0.001 |
| Women ^c^ | -7.9 | <0.001 |
| **Subjects without statin medication or CHD** | | |
| Metabolic syndrome ^cd^ | -0.93 | <0.001 |

^a^ see section 2.2.6 for details; ^b^ model: HDL2/HDL ratio = B_1_ x early CHD + intercept (age-matched groups are compared in men only); ^c^ model: HDL2/HDL ratio = B_1_ x MetS + B_2_ x age + intercept; ^d^ HDL2/HDL ratio is transformed to z-scores in sexes separately to adjust for sex.
