# Supplementary figures and images for: Impaired HDL2-mediated cholesterol efflux is associated with metabolic syndrome in families with early onset coronary heart disease and low HDL-cholesterol level
Source: PLoS One. 2017 Feb 16;12(2):e0171993. doi: 10.1371/journal.pone.0171993 (PMC5313225; doi:10.1371/journal.pone.0171993)

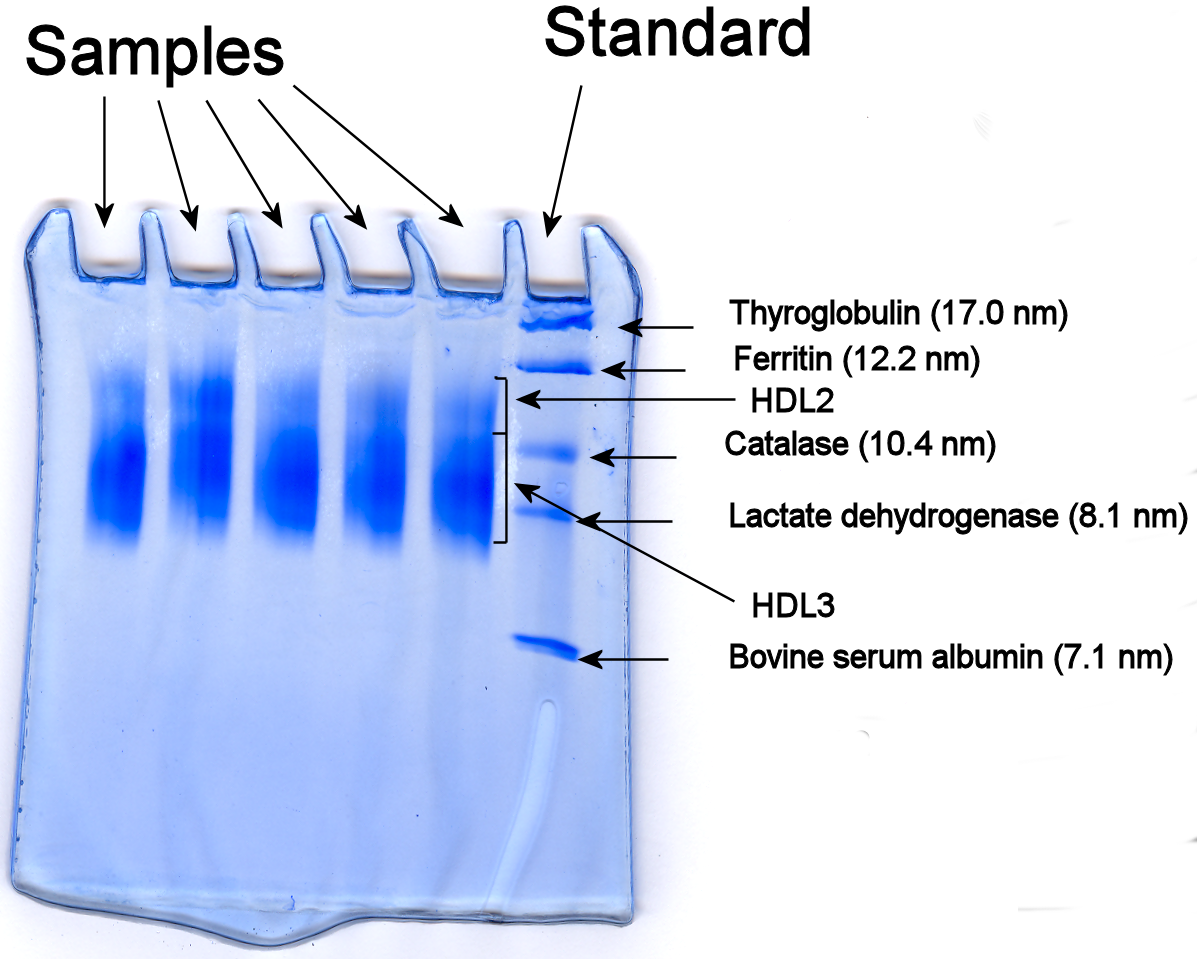

Supplement: S1 Fig — An exemplary image of five samples. (PNG) [file pone.0171993.s004.png]
